# Flavonoids from Sacred Lotus Stamen Extract Slows Chronological Aging in Yeast Model by Reducing Oxidative Stress and Maintaining Cellular Metabolism

**DOI:** 10.3390/cells11040599

**Published:** 2022-02-09

**Authors:** Duangjai Tungmunnithum, Samantha Drouet, Christophe Hano

**Affiliations:** 1 Department of Pharmaceutical Botany, Faculty of Pharmacy, Mahidol University, Bangkok 10400, Thailand; 2 Laboratoire de Biologie des Ligneux et des Grandes Cultures, INRAE USC1328, Campus Eure et Loir, Orleans University, 28000 Chartres, France; samantha.drouet@univ-orleans.fr; 3 Le Studium Institue for Advanced Studies, 1 Rue Dupanloup, 45000 Orléans, France

**Keywords:** sacred lotus stamen extract, *Nelumbo nucifera*, stamen extract, flavonoids, molecular mechanisms, antiaging, chronological aging

## Abstract

*Nelumbo nucifera* is one of the most valuable medicinal species of the Nelumbonaceae family that has been consumed since the ancient historic period. Its stamen is an indispensable ingredient for many recipes of traditional medicines, and has been proved as a rich source of flavonoids that may provide an antiaging action for pharmaceutical or medicinal applications. However, there is no intense study on antiaging potential and molecular mechanisms. This present study was designed to fill in this important research gap by: (1) investigating the effects of sacred lotus stamen extract (LSE) on yeast lifespan extension; and (2) determining their effects on oxidative stress and metabolism to understand the potential antiaging action of its flavonoids. A validated ultrasound-assisted extraction method was also employed in this current work. The results confirmed that LSE is rich in flavonoids, and myricetin-3-*O*-glucose, quercetin-3-*O*-glucuronic acid, kaempferol-3-*O*-glucuronic acid, and isorhamnetin-3-*O*-glucose are the most abundant ones. In addition, LSE offers a high antioxidant capacity, as evidenced by different in vitro antioxidant assays. This present study also indicated that LSE delayed yeast (*Saccharomyces cerevisiae*, wild-type strain DBY746) chronological aging compared with untreated control yeast and a positive control (resveratrol) cells. Moreover, LSE acted on central metabolism, gene expressions (*SIR2* and *SOD2*), and enzyme regulation (SIRT and SOD enzymatic activities). These findings are helpful to open the door for the pharmaceutical and medical sectors to employ this potential lotus raw material in their future pharmaceutical product development.

## 1. Introduction

*Nelumbo nucifera* Gaertn. is Asian lotus species that belongs to the family Nelumbonaceae [1,2,3,4]. *N. nucifera* is also well known as sacred lotus, and its major distribution is in Thailand, India, China, and many Asian countries [1,2,3,5,6]. Almost every part/organ of the sacred lotus has long been used as food, herbal supplements, or herbal medicines, especially in Thailand, China, and India [2,3,4,7,8,9]. Stamen is the most valuable part of sacred lotus, and is employed in several traditional medicines or herbal drugs for relaxation and health benefits, such as boosting the immune system and improving blood circulation [4,10,11]. According to the preparation of various traditional medicines; for example, Chinese traditional medicines, Kampo medicines (the traditional Japanese herbal medicines), and Thai traditional medicines, these raw plant materials are commonly used in the form of an extract [12,13,14,15], following the traditional knowledge that the extract, which consists of major bioactive compounds, will provided synergistic effects for the health benefits of mankind. Nowadays, the number of research studies on the potential pharmacological activities of *N. nucifera* are considerably increased [7,8,9,10,11,16,17,18,19,20].

Aging is a multifactorial biological process that is influenced by a range of hereditary and/or environmental variables [21]. A variety of theories have been proposed to explain the aging process, the most well known being the free radical theory of aging introduced in 1956 by Denham Harman, which remains a viable explanation for the aging process [22,23]. According to the idea, aging is caused by accumulated oxidative stress, which causes oxidative damage to vital biomolecules (including membrane lipids, proteins, or DNA) within the cell, leading to functional declines, cellular aging, and possibly death [22]. As a result, antioxidants that can scavenge reactive oxygen species and/or reactive nitrogen species (ROS and RNS, respectively) may be useful in slowing the aging process. Plant-derived antioxidants, particularly polyphenols, have been demonstrated to offer therapeutic promise for aging and age-related illnesses in several investigations [22,24,25,26,27]. Resveratrol and other plant polyphenols have been shown to extend the lifespan of numerous species, working through a well-conserved mechanism, initially observed in yeast, and later in other models including insect (*Drosophila melanogaster*), nematode (*Caenorhabditis elegans*), and mammal (mouse) [24,25,26,27,28,29]. Yeast has proven to be a useful model in the search for antiaging chemicals [30,31,32], due to the high degree of gene conservation and aging mechanisms shared between yeast and humans [30,33]. The successful discovery of prospective antiaging substances following their preliminary examination in yeast is therefore not unexpected [24,26,27,29,30].

In particular, an antiaging plant polyphenol, resveratrol, was initially found to postpone or attenuate aging by activating silent information regulator 2 (SIR2) in a budding yeast model [24]. Other genetic models [28,29] have confirmed resveratrol’s action on sirtuins (conserved orthologs of the *sir2* gene). In yeast, SIR2 activation by resveratrol has been observed at both the gene expression and enzyme activation levels [34,35,36,37]. However, the mechanism behind the potential lifespan benefits of resveratrol and other plant polyphenols is yet unknown [38]. Sirtuins are a family of nicotinamide adenine dinucleotide (NAD)-dependent protein deacetylases that have been connected to ROS production and aging, particularly the ROS-driven mitochondria response [39].

Other antiaging plant polyphenols, as well as their molecular mechanisms of action, should be studied. Such research might yield valuable information for the usage and development of antiaging plant sources and derived compounds, as well as disclose pathways that could lead to the creation of antiaging drugs. The potential antiaging effect of sacred lotus (*N. nucifera*), in particular against degenerative diseases, has been recently ascribed to its flavonoid fraction [40]. In lotus species, the stamen is an enriched source of flavonoids [4,41]. The current study investigated the effects of *N. nucifera* stamen extract on yeast lifespan extension, as well as its effects on oxidative stress and metabolism, to extend our understanding of the potential antiaging action of its flavonoids.

## 2. Materials and Methods

### 2.1. Chemicals and Reagents

All solvents and reagents used in the extraction and high-performance liquid chromatography (HPLC) analyses were of analytical grade or the purest available grade (Thermo Fisher Scientific, Illkirch, France). A Milli-Q water-purification system was used to prepare pure deionized water (Merck Millipore Fontenay sous Bois, Paris, France). Before utilization, all solutions that were produced for HPLC analysis were filtered through 0.45 m nylon syringe membranes. Standard compounds were purchased from Extrasynthese (Genay, France).

### 2.2. Plant Materials

The living plant specimens of *N. nucifera* were collected from a natural habitat in Nakhon Ratchasima, Thailand. The plant samples were then identified at the species level by using the taxonomic keys and species description in the existing Floras [1,2], and also were compared with herbarium specimens kept at the Forest Herbarium (BKF), Bangkok, Thailand; and at the Professor Kasin Suvatabandhu Herbarium, Chulalongkorn University, (BCU) Bangkok, Thailand. The herbarium abbreviations were used following the Thiers team [42]. The plant samples were then air-dried and prepared following the recommendations of the World Health Organization [43].

### 2.3. Extraction

First, 100 mg of dried stamens of *N. nucifera* were poured into 5 mL quartz tubes coupled with a vapor condenser, mixed with 1 mL 90% (*v*/*v*) aqEtOH, and extracted using an USC1200TH ultrasonic bath (Prolabo, Fontenay-sous-Bois, France) following the optimum extraction conditions: 45 min, 45 °C, 30 kHz frequency [41]. After that, the extract was centrifuged at 5000× *g* for 15 min (Heraeus Biofuge Stratos, Thermo Scientific, Illkirch, France), and the supernatant was filtered using 0.45 µm nylon syringe membranes (Merck Millipore, Saint-Quentin Fallavier, France). The flavonoid enrichment was achieved through the previously published extra DAX-8 macroporous resin purification step (Merck Millipore, Saint-Quentin Fallavier, France) [41].

### 2.4. HPLC Analysis

The HPLC system, composed of an autosampler, a Prostar 230 pump, and a Prostar 335 photodiode array detector, operated using the Galaxie software (Varian v1.9.3.2) (Varian, Les Ulis, France). The separation was then performed at 40 °C with a Purospher RP-18 column (250 × 4.0 mm internal diameter; 5 µm) (Merck Chemicals, Molsheim, France). The mobile phase was made up of a combination of methanol (solvent A) and HPLC-grade water (solvent B), both of which were acidified with 0.05% (*v*/*v*) formic acid. The mobile phase was varied using a linear gradient, ranging from 5:95 (*v*/*v*) to 100:0 (*v*/*v*) mixes of solvents A and B, respectively, at a flow rate of 1.30 mL/min. The injection volume was 3 µL, the maximum back pressure was 110 bar, and the detecting wavelength was 320 nm. The flavonoid compounds were then identified by comparing with the authentic standard compounds (Sigma Aldrich, Saint-Quentin Fallavier, France).

### 2.5. In Vitro Cell Free Antioxidant Assays

For the in vitro antioxidant assays, ABTS (2,2-azinobis(3-ethylbenzthiazoline-6-sulfonic acid), DPPH (2,2-diphenyl-1-picrylhydrazyl) and ferric-reducing antioxidant power (FRAP) assays were employed to determine the antioxidant effect of the extracted samples following the adapted protocols from the microplate reader (Multiskan GO, Thermo Fisher Scientific, Illkirch, France), as previously described [41,44].

### 2.6. Yeast Culture Conditions

The wild-type yeast *Saccharomyces cerevisiae* strain DBY746 (*MATα leu*2-3,112 *his*3Δ1 *trp*1-289a *ura*3-52 GAI^+^, Yeast Genetic Stock Center, Manassas, VA, USA) was employed in this study. The culture was initiated from frozen stock plated onto a yeast extract peptone dextrose medium (YPD medium) (Sigma-Aldrich, Saint-Quentin Fallavier, France).

### 2.7. Chronological Aging Evaluation

Lifespan was evaluated as follows: after the incubation for 2–3 days at 30 °C, a single colony was then incubated in 1 mL of synthetic complete dextrose medium (SDC medium) [45] and then incubated overnight with shaking at 30 °C and 220 rpm. The overnight culture was then diluted into 10 mL of new SDC medium to an absorbance value of 600 nm of 0.1, and incubated at 30 °C, with shaking at 220 rpm. This time point was considered Day 0 of chronological aging. A yeast chronological lifespan (CSL) experiment in liquid culture was investigated, as reported by Hu et al. [45]. In brief, aliquots of 10 µL were taken from the flask and diluted 10,000 times with sterile water. The diluted culture was then placed on YPD plates and cultured for 3 days at 30 °C, after which the colony-forming unit (CFU) counts were calculated. Under a light microscope, the microcolonies growing on the YPD plates were spotted, and the daughter cells were counted. The nontreated cells’ CFU number at Day 3 was calculated to represent 100% survival. The lotus stamen extract (LSE) and resveratrol used as positive control (at 10 µM final concentration) were then dissolved in dimethyl sulfoxide (DMSO cell culture grade; Sigma-Aldrich, Saint-Quentin Fallavier, France). The final concentration of DMSO was 0.1% (*v*/*v*). The control yeast was inoculated with a similar concentration of DMSO.

An absorbance ratio of 600 nm of the yeast cultures measured at 0 and 48 h after treatment was used to calculate the growth index [46].

### 2.8. Reactive Oxygen and Nitrogen Species Measurement

Dihydrorhodamine-123 (DHR-123) fluorescent dye (Sigma-Aldrich, Saint-Quentin Fallavier, France) was employed to evaluate the reactive oxygen and nitrogen species (ROS/RNS) production levels as described previously [47]. For this, ca. 10^8^ yeast cells were washed twice using the PBS, resuspended in the PBS with 0.4 μM of DHR-123, and then incubated for 10 min at 30 °C in the dark. After performing the washing step 2 times using the PBS, the fluorescence signal (λex = 505 nm and λem = 535 nm) was then detected by using the VersaFluor Fluorimeter (Biorad, Marnes-la-Coquette, France).

### 2.9. Estimation of NAD, NADH, and ATP Contents

The NAD and NADH measurements were performed as described previously [48] using ca. 10^7^ cells. Briefly, after the extraction of NAD and NADH using the acidic and alkali extraction protocol, the concentrations were determined fluorometrically with the excitation at 365 nm and the emission at 460 nm by using the VersaFluor Fluorimeter (Biorad, Marnes-la-Coquette, France) with the standard curves (0–40 µM).

ATP was quantified as described previously [49] using the colorimetric ATP Assay Kit (Merck Millipore, Saint-Quentin Fallavier, France) according to the manufacturer’s instructions.

### 2.10. Mitochondrial Membrane Potential and Metabolic Activity Evaluation

The mitochondrial membrane potential (ΔΨm) was determined by using the monitoring fluorescence of the specific probe 3,3′-dihexyloxacarbocyanine iodide (DiOC6(3); (Sigma-Aldrich, Saint-Quentin Fallavier, France) as described previously [50]. DiOC6(3) differentially stained mitochondria as a function of their ΔΨm [51]. The cells were then incubated in the culture medium with 25 nM of DiOC6(3) at 30 °C for 45 min. After that, their fluorescence signal (λex = 482 nm and λem = 504 nm) was determined using the VersaFluor Fluorimeter (Biorad, Marnes-la-Coquette, France). The results were then expressed in the form of relative fluorescent units.

The metabolic activity of yeast cells was evaluated using the FUN-1 probe according to the manufacturer’s instructions (Molecular Probes, Illkirch, France) with previously described modifications [52].

### 2.11. Gene Expression by RT-qPCR Analysis

The total RNAs from yeast cells were extracted during their exponential phase by using the RiboPure RNA extraction kit (Thermo Scientific, Illkirch, France). The reverse transcription was then done by using the SuperScript IV cDNA synthesis kit (Thermo Scientific, Illkirch, France) with the oligodT primer (Thermo Scientific, Illkirch, France), 1 unit of RiboLock RNAse inhibitor (Thermo Scientific, Illkirch, France), and 5 mg of yeast total RNA obtained following quantification using a Quant-iT HR RNA assay, and using a Qubit fluorimeter (Thermo Scientific, Illkirch, France). The real-time PCR was then done using a PikoReal^TM^ Real-Time PCR System (Thermo Scientific, Illkirch, France) with DyNAmo ColorFlash SYBR Green qPCR (Thermo Scientific, Illkirch, France), as well as the specific primers.

The primers used in this study were:

- *sod1*, forward: 5′-CACCATTTTCGTCCGTCTTT-3′, and reverse: 5′-TGGTTGTGTCTCTGCTGGTC-3′;
- *sod2*, forward: 5′-CTCCGGTCAAATCAACGAAT-3′, and reverse: 5′-CCTTGGCCAGAAGATCTGAG-3′;
- *sir2*, forward: 5′-CGTTCCCCAAGTCCTGATTA-3′, and reverse: 5′-CCACATTTTTGGGCTACCAT-3′;
- *ndi-1*, forward: 5′-GGTGGTGGGCCTACTGGTGT-3′, and reverse: 5′-TTCAAAACGATGGGCAGAGC-3′;
- *tub1*, forward: 5′-CCAAGGGCTATTTACGTGGA-3′, and reverse: 5′-GGTGTAATGGCCTCTTGCAT-3′.

The qPCR parameters were: (1) 5 min, 95 °C, (2) 40 three-step cycles for 15 s, 94 °C; 10 s, 55 °C; and 20 s, 72 °C. A final extension phase was performed at 72 °C for 90 s. The observation of a single peak in the melting curve obtained after amplification showed the existence of a single amplicon. The amounts of the *sir2*, *sod1*, *sod2*, and *ndi-1* mRNA were normalized to that of the *tub1*. The expression levels were then calculated and normalized using the 2^−ΔΔCt^ method.

### 2.12. Enzymatic Activities Determinations

For the experiment of protein extraction, approximately 10^8^ yeast cells were washed 3 times using the PBS. After that, 1 mL of the PBS was added, then the mixture was subjected to 3 freeze–thaw cycles using liquid nitrogen. The cell lysate was centrifuged at 10,000× *g* for 15 min at 4 °C, and the supernatant was then used to prepare the sample solution. The proteins were then quantified using a Qubit Protein Assay Kit according to instructions from the manufacturer, and using a Qubit fluorimeter (Thermo Scientific, Illkirch, France).

The total SOD activity was examined using a Superoxide Dismutase Activity Kit according to instructions from the manufacturer (Thermo Scientific, Illkirch, France).

The SIRT1/SIR2 activity was then investigated by using a SIRT1 Assay Kit (Sigma-Aldrich, Saint-Quentin Fallavier, France) according to instructions from the manufacturer, and using a Versafluor fluorimeter (Biorad, Marnes-la-Coquette, France).

Total NADH oxidase activity was assayed spectrophometrically at 25 °C in 50 mM of potassium phosphate buffer (pH 7.0), 0.29 mM NADH, and 0.3 mM EDTA [53]. A unit of activity was the quantity that catalyzed the oxidation of 1 μmol of NADH per min.

### 2.13. Membrane Lipid Peroxidation Evaluation

A thiobarbituric acid (TBA; Sigma Aldrich, Saint-Quentin Fallavier, France) method previously reported [51] was used for measurement of the membrane lipid peroxide. In brief, approximately 10^8^ yeast cells ground in the double-distilled water were centrifuged at 10,000× *g* for 10 min. Then, 75 μL of supernatant was mixed with 25 μL of SDS (3% (*w*/*v*)), 50 μL of TBA (3% (*w*/*v*) prepared in 50 mM NaOH), and 50 µL HCl (23% (*v*/*v*)). Mixing was carried out between each addition. The final mixture was heated at 80 °C for 20 min, and then cooled on ice. The absorbance was determined at 532 nm (A532). The nonspecific absorbance determined at 600 nm (A600) was subtracted. The standard curve was then prepared by using 1,1,3,3-tetramethoxypropane to determine the concentrations of TBARS in the samples.

### 2.14. Protein Carbonylation Level Estimation

The total proteins were extracted from about 10^8^ yeast cells following the method given in Section 2.12 The protein carbonyl content was determined using the Protein Carbonyl ELISA kit according to the manufacturer’s instructions (Cell BioLabs, Paris, France).

### 2.15. 8-Oxo-Guanine Level Estimation

The DNA was extracted from about 10^8^ yeast cells with a Yeast DNA Extraction Reagent Kit according to the manufacturer’s instructions (Thermo Scientific, Illkirch, France). The 8-oxo-guanine content was then examined by the 8-OHdG DNA Damage ELISA Kit according to the manufacturer’s instructions (Cell BioLabs, Paris, France).

### 2.16. Statistical Analysis

The results were expressed in the form of the means and the standard deviations of at least 4 separated (biologically independent) replicates employed to show the data. The significant differences between the groups in all the experiments were investigated using ANOVA, followed by 2-tailed multiple *t*-tests with a Bonferroni correction performed using the XL-STAT 2019 biostatistics software (Addinsoft, Paris, France). All these results were considered significantly different at *p* < 0.05.

## 3. Results and Discussion

### 3.1. LSE (Sacred Lotus Stamen Extract) Is Rich in Antioxidant Flavonoids

**Figure 1:**
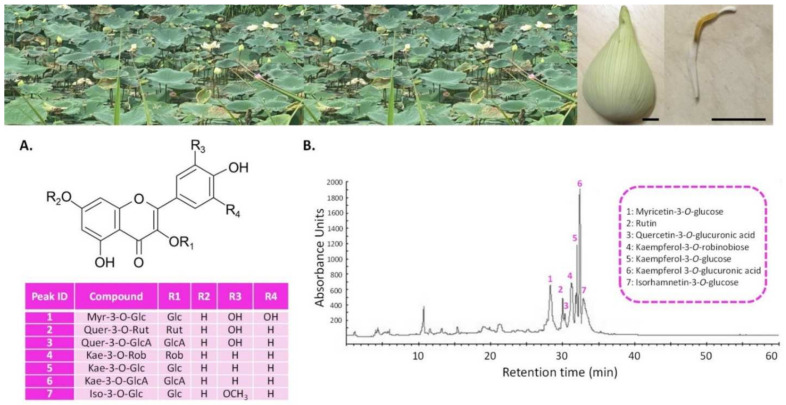
(**A**) Pictures of sacred lotus’s habitat, perianth, and stamen; bar = 1 cm; (**B**) Structures and corresponding numbers on the HPLC chromatogram of the main phenolic compounds considered in this study: **1**. Myr-3-*O*-Glc: myricetin-3-*O*-glucose; **2**. Quer-3-*O*-Rut: quercetin-3-*O*-rutinoside (rutin); **3**. Quer-3-*O*-GlcA: quercetin-3-*O*-glucuronic acid; **4**. Kae-3-*O*-Rob: kaempferol-3-*O*-robinobiose; **5**. Kae-3-*O*-Glc: kaempferol-3-*O*-glucose; **6**. Kae 3-*O*-GlcA: kaempferol 3-*O*-glucuronic acid; **7**. Iso-3-*O*-Glc: isorhamnetin-3-*O*-glucose. HPLC chromatogram (detection set at 320 nm) of the sacred lotus stamen extract (LSE) prepared with an ultrasound-assisted extraction method, followed by flavonoid enrichment using the moderately polar acrylic DAX8 macroporous resin procedure. The mean and standard deviation of the concentration of each flavonoid is provided in Table S1. All figures by C.H. and D.T.

The sacred lotus (*N. nucifera*) stamen extract (LSE) used in this study was prepared using a validated ultrasound-assisted extraction method, followed by flavonoid enrichment using the moderately polar acrylic DAX8 macroporous resin procedure [41]. The HPLC chromatogram and the detailed phytochemical analysis of this extract are shown in Figure 1 and Table S1, respectively.

This analysis confirmed that the present extract was rich in flavonoids, with myricetin-3-*O*-glucose, quercetin-3-*O*-glucuronic acid, kaempferol-3-*O*-glucuronic acid, and isorhamnetin-3-*O*-glucose being the most abundant. This extract presented a high antioxidant capacity, as evidenced by different in vitro antioxidant assays (Table S2). It was described in the literature that, compared to other organs, lotus stamen extract has a stronger antioxidant capacity, which was largely attributed to its high flavonoid concentration [3,10,40]. As a consequence, the present results were in line with these reports, particularly that of Jung et al. [10], who reported the strong radical scavenging capacity of kaempferol glycosides from lotus stamen. More importantly, this demonstrated that the presented LSE was bioactive.

We monitored the chronological aging of wild-type yeast (*Saccharomyces cerevisiae*, strain DBY746) previously employed for this purpose in order to examine the possible action of the LSE [32,54]. When grown on SDC medium, this wild-type strain has a short mean lifespan [54], which is useful for evaluating the effect of plant extracts on the yeast lifespan. To minimize any bias arising from putative toxic and/or antifungal actions, the lack of any significant growth and viability impacts of LSE were examined at the varied doses prior to the determination of its influence on lifespan (Table S2). The different concentrations examined showed no signs of toxicity (Table S2). We adopted the application of the LSE at a final concentration of 0.5 mg/mL, which is the most commonly used concentration for the evaluation of lotus extract health benefits [55].

### 3.2. LSE Delays Yeast Chronological Aging

**Figure 2:**
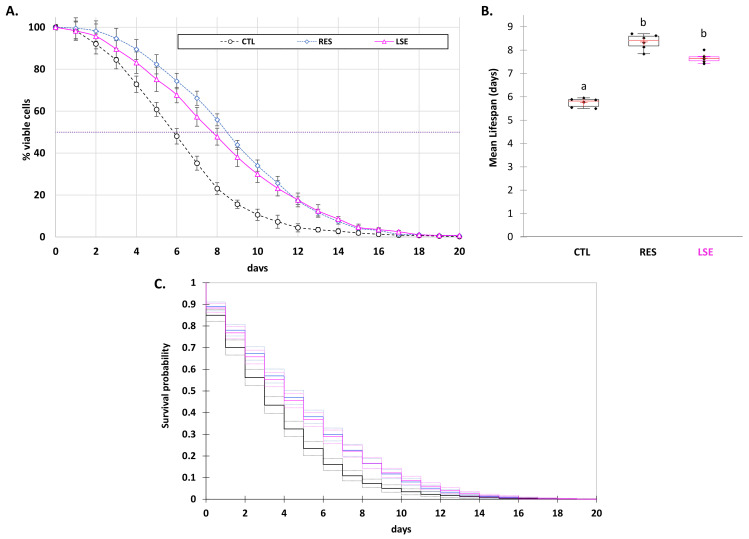
(**A**) Viability plot of yeast (strain DBY746) determined using the microcolony method on yeast extract peptone dextrose (YPD) plates. (**B**) Mean of chronological lifespan yeast (strain DBY746) calculated using the viability plot. Significant differences (*p* < 0.05) are shown by different letters. (**C**) Kaplan–Meier estimator showing the survival function from the viability plot. The values represent the means and standard deviations of six independent experiments. LSE: sacred lotus stamen extract (0.5 mg/mL); RES: E-resveratrol (10 µM) used as positive antiaging control.

Figure 2 shows the results obtained with LSE in the yeast (*Saccharomyces cerevisiae*) wild-type DBY746 strain chronological aging assay, which were compared to yeast control (inoculated with the same volume of DMSO) and resveratrol (RES) employed as a positive control (applied at the commonly used concentration of 10 µM) [32,52,56,57]. The yeast DBY746 strain was the model used to study the chronological lifespan [54]. As previously described [32,52,56,57], the impact on chronological lifespan was evaluated using the cellular viability lifespan of yeast measured as the yeast population survival (Figure 2A), allowing us to determine the mean lifespan (Figure 2B), as well as using the Kaplan–Meier estimator, which allowed us to determine the mean survival probability (Figure 2C) [58].

The mean lifespan of the CTL DBY746 yeast matured in the SDC was significantly increased by LSE (7.66 ± 0.11 days for LSE vs. 5.75 ± 0.24 days for the control yeast cell). Moreover, the impact of LSE did not vary significantly from that of resveratrol (RES, 8.36 ± 0.24 days), the standard positive control for evaluating the extension of chronological lifetime (Figure 2A,B). The use of the Kaplan–Meier estimator also supported this tendency, with LSE-treated yeast cells having a higher mean survival time probability (4.78 ± 0.12 days) than control yeast cells (3.60 ± 0.12 days), which was similar to the action of resveratrol (4.74 ± 0.13 days) on yeast lifespan extension (Figure 2C). The standard chronological aging approach was a suitable model for studying the aging of nondividing cells in higher eukaryotes, including humans, since it allowed for a direct assessment of postmitotic cell aging. Yeast has proven to be a useful tool in the search for antiaging compounds [30,31]. It has been demonstrated that antioxidant polyphenols such as resveratrol and flavonoids extend the longevity of a variety of higher eukaryotes such as yeast, Caenorhabditis elegans, Drosophila melanogaster, and mice via common processes [59]. The most notable example is indisputably the antiaging activity of resveratrol, originally demonstrated in yeast and afterward in other models [24,28,29,33]. The present results clearly indicated that LSE statistically and substantially extended the lifespan of the yeast cells. This could be due to its high concentration of antioxidant flavonoid.

### 3.3. LSE Protects Cells from Oxidative Stress Damage by Maintaining Mitochondrial Functions

**Figure 3:**
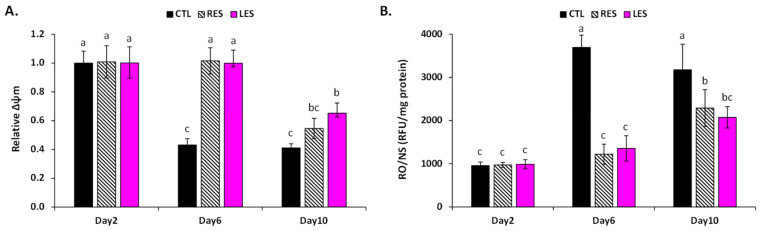
(**A**) Mitochondria integrity estimated by mitochondrial potential (ΔΨm) variation; (**B**) production of reactive oxygen and nitrogen species (ROS/RNS) during aging evaluated at 3 different times (2, 6, and 10 days of culture). The evaluation of mitochondria membrane potential (ΔΨm) was carried out by treating cells with 3,30-dihexyloxacarbocyanineiodide (DiOC6(3). ROS/RNS production was evaluated using the dihydrorhodamine-123 (DHR123) probe. LSE: sacred lotus stamen extract (0.5 mg/mL); RES: E-resveratrol (10 µM) used as positive antiaging control. Significant differences (*p* < 0.05) are shown by different letters.

**Figure 4:**
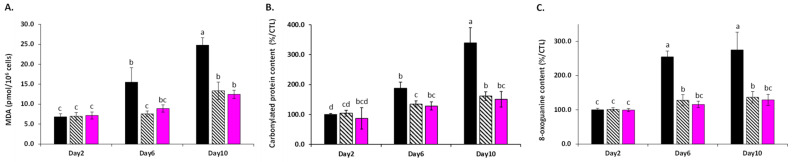
Production of: (**A**) malondialdehyde (MDA, indicators of membrane lipid peroxidation) concentrations determined by the TBARS assay; (**B**) protein carbonyl contents determined by ELISA assay (as a biomarker of oxidative damages on proteins); (**C**) 8-oxo-guanine content determined by ELISA assay (as an indicator of oxidative damages at DNA level). LSE: sacred lotus stamen extract (0.5 mg/mL); RES: E-resveratrol (10 µM) used as positive antiaging control. Significant differences (*p* < 0.05) are shown by different letters.

To estimate the relationship between the antioxidant capacity of the LSE and its ability to extend the yeast’s chronological lifespan, LS’s in vivo antioxidant capacity was evaluated by measuring reactive oxygen and nitrogen species (ROS/RNS) production, induced oxidative damages, and mitochondrial functionality during aging (Figure 3 and Figure 4).

During aging, in control cells, a possible loss of mitochondrial functions was assessed by the disruption of the mitochondrial membrane potential (ΔΨm). On the contrary, both LSE- and resveratrol-treated cells were able to keep a functional ΔΨm value for a longer period of time, and hence may have been able to maintain normal mitochondrial functions (Figure 3A). Mitochondria are the core of oxidative metabolism and the main producers of reactive oxygen and nitrogen species (ROS/RNS), which are physiologically and continuously generated as byproducts of aerobic activity in living organisms [60]. As a result of mitochondrial dysfunction, ROS/RNS production increased with age. This is the fundamental basis of the free radical theory of aging [22,23], which claims that aging is induced by cumulative oxidative stress that causes oxidative damages to various macromolecules (including membrane lipids, proteins, and DNA) within the cell, leading to cell disfunction and possibly death [22]. In accordance with this theory, the production of ROS/RNS was considerably higher in the aging control yeast (Figure 3B). In contrast, unlike resveratrol-treated cells, ROS/RNS formation increased only moderately in LSE-treated cells during aging (Figure 3B). These results indicated that LSE may operate by modulating the ROS/RNS aging production mediated by mitochondria.

The effect of this ROS/RNS aging formation mediated by mitochondria on macromolecules was next assessed by measuring the levels of peroxidized membrane lipids, carbonylated proteins, and oxidized DNA (Figure 4). Overall, the results indicated that both LSEs provided excellent protection against a wide range of oxidative-stress-induced damages. Indeed, as compared to the aging control cells, the levels of malondialdehyde (MDA), protein carbonyl, and 8-oxo-guanine (which are biomarkers of oxidative damages to lipid membranes, proteins, and DNA, respectively) remained comparatively low. Here, the use of resveratrol as a positive control also supported this antioxidative action. Indeed, several plant extracts and/or phytochemicals, including resveratrol, have been shown to prolong lifespan in yeast by decreasing oxidative stress and its damages [34,35]. As a result, the wide antioxidant action may be connected to ability of LSE to extend yeast lifespan.

Although this antioxidant component appeared to be relevant, it is possible that it is not the only one to be regarded in explaining the LSE effect. Mitochondria are the major centers of cellular metabolism and energy production. Furthermore, protein carbonyl groups have been found to impact the protein structure [61] and, as a result, protein functions (which could be involved in various processes, such as a metabolic sequence in the case of an enzyme, or the gene expression regulation in the case of a transcription factor or important regulator). Our next goal was to examine this possibility by tracking LSE’s effect on the central metabolism and the regulation of key genes and enzymes involved in the aging process.

### 3.4. LSE Acts on Central Metabolism, Gene Expressions, and Enzyme Regulation

**Figure 5:**
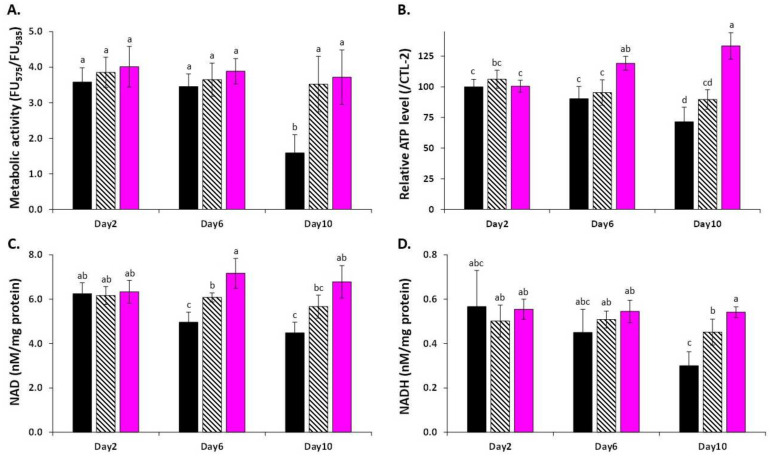
Impact of LSE on central metabolism. (**A**) metabolic activity determined using the FUN-1 probe, (**B**) ATP production, (**C**) NAD level, and (**D**) NADH level. LSE: sacred lotus stamen extract (0.5 mg/mL). RES: E-Resveratrol (10 µM) used as positive antiaging control. Significant differences (*p* < 0.05) are shown by different letters.

Here, LSE-treated yeast cells maintained their metabolic activity, as evidenced by the JUN-1 probe, but also cellular ATP content and NAD and NADH levels (Figure 5).

It is worth noting that the cellular ATP content and NAD level were both enhanced, at least transiently, in LSE-treated cells compared to both control and resveratrol-treated cells. However, the NAD/NADH ratio remained stable for each treatment (Figure S1). It has been shown that caloric restriction significantly increased ATP levels and has been linked to the extension of lifespan in yeast [49]. Similarly, NAD levels have been related to the extension of replicative lifespan in yeast [62]. NAD is the cofactor of SIR2, and therefore is essential for its function [63]. SIR2 is a member of the NAD-dependent protein deacetylase family, and its activity has been linked to ROS and aging, particularly the ROS-driven mitochondrial-mediated response [39]. Through stimulation of SIR2 activity, resveratrol was one of the first plant polyphenols to postpone yeast aging [24]. SIR2 is also involved in lifespan extension induced by calorie restriction [63], thus indicating a possible relation with central metabolism regulation.

**Figure 6:**
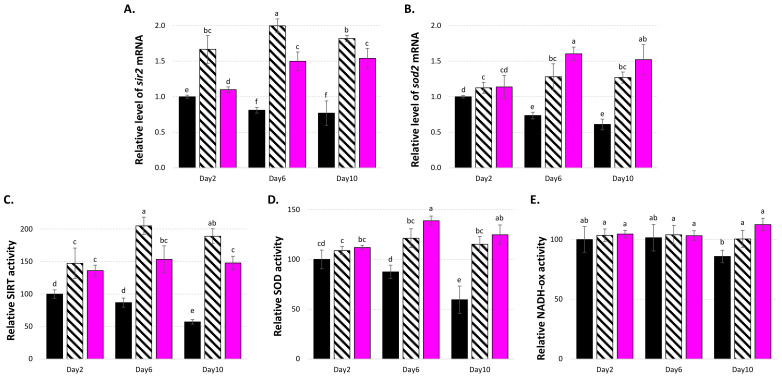
Effects of LSE on: (**A**) *sir2* and (**B**) *sod2* gene expression; (**C**) SIRT1; (**D**) SOD; and (**E**) NADH oxidase enzyme activities. LSE: sacred lotus stamen extract (0.5 mg/mL); RES: E-resveratrol (10 µM) used as positive antiaging control. Significant differences (*p* < 0.05) are shown by different letters.

In line with these observations, LSE was demonstrated to stimulate *sir2* and *sod2* gene expression (Figure 6A,B), as well as their respective SIRT and SOD enzymatic activities (Figure 6C,D). In comparison to young yeast cells, aged yeast cells presented decreased expression of the *sir2* and *sod2* genes, although the positive control resveratrol reversed this tendency, as already observed [56,57,64,65].

Superoxide dismutases (SODs) are antioxidative enzymes involved in ROS scavenging that are encoded in yeast by two genes: *sod1*, encoding for a Cu/Zn-SOD found in the cytoplasm; and *sod2*, encoding for a Mn-SOD found in the mitochondria. SOD2 is an excellent ROS scavenger that plays a key role in antioxidant response [66] and has been linked to yeast lifespan regulation [67]. Here, the enhanced SOD enzyme activity may have been related to the *sod2* gene expression activation, since *sod1* gene expression was less affected by LES treatment (Figure S2). SIRT/SIR2 has been shown in various cellular and animal models to promote *sod2* gene expression via a deacetylation mechanism that activates both PGC-1 and FOXO [68,69]. This resulted in the stimulation of *sod2* gene expression by diverse plant-derived natural compounds in yeast, which was linked to a longer lifespan [34,35,36]. In the present study, the maintenance of a functional ΔΨm value may have been connected to increased *sod2* gene expression and, as a result, higher SOD activity in the current investigation. Furthermore, the increased NAD level (therefore SIRT action) and higher ATP content may have been linked to the stimulation of NADH oxidase enzyme activity seen in LSE-treated cells (Figure 6E). Thus, altogether these results indicated that the effects of LSE may be connected to a more complex process involving the maintenance of normal mitochondrial functioning, particularly metabolic functions, rather than just a simple antioxidant activity, and that LSE might be an interesting candidate for use in dietary natural products for the treatment of oxidative and age-related diseases.

## 4. Conclusions

**Figure 7:**
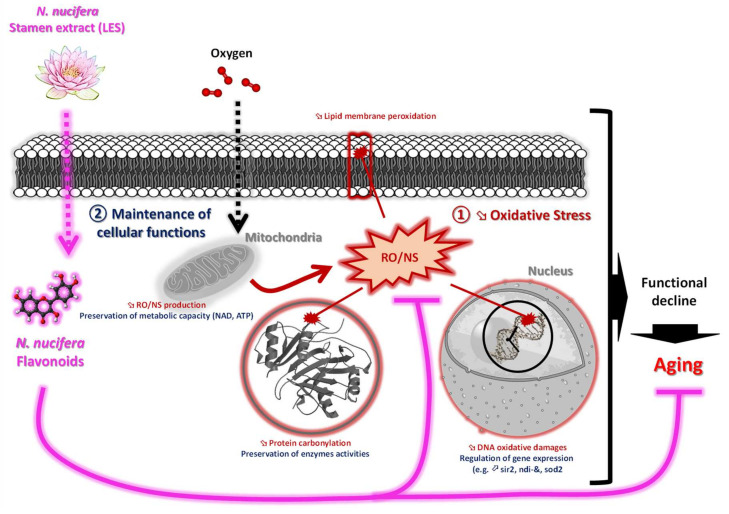
Simplified view summarizing the effects of LSE on chronological aging in yeast.

To sum up, the present study pointed out the potential of sacred lotus stamen extract as a rich antioxidant flavonoid raw material that offers antiaging potential to delay yeast chronological aging by acting both at the gene expression (*sir2* and *sod2*) and enzyme regulation levels, leading to: (1) a reduction in oxidative stress by reducing ROS/RNS production and their induced oxidative damages through the control of essential antioxidant enzymes such as SOD2; as well as (2) the maintenance of central metabolism, passing in particular through the maintenance of functional mitochondrial functions, allowing longer maintenance of efficient NAD and ATP production. The highlight of this major finding is summarized in Figure 7. Altogether, these findings provided frontier results that will be helpful to both the pharmaceutical and medical sectors in considering sacred lotus stamen extract as a potential alternative starting plant material for antiaging drug development or other pharmaceutical products with antiaging benefits. However, a toxicity test and clinical trials should be conducted to validate the safety and efficacy of any future developed products.

## Data Availability

All the data supporting the findings of this study are included in this article.

## Supplementary Materials

The following supporting information can be downloaded at: https://www.mdpi.com/article/10.3390/cells11040599/s1, Figure S1. Impact of LSE on NAD/NADH ratio. LSE: sacred lotus stamen extract (0.5 mg/mL); RES: *E*-Resveratrol (10 µM) used as positive antiaging control. Significant differences (*p* < 0.05) are shown by different letters; Figure S2. Effects of LSE on (A) *SOD1* and (B) *NDI-1* gene expression. LSE: sacred lotus stamen extract (0.5 mg/mL); RES: E-resveratrol (10 µM) used as positive antiaging control. Significant differences (*p* < 0.05) are shown by different letters; Table S1. Quantification of the major flavonoids of the *N. nucifera* stamen extract; Table S2. In vitro antioxidant capacity of the *N. nucifera* stamen; Table S3. Viability of yeast cells under the different treatment conditions determined 48 h after treatment.

## Sample Availability

Samples of the compounds are available from the authors.
