# Supplementary material for: Flavonoids from Sacred Lotus Stamen Extract Slows Chronological Aging in Yeast Model by Reducing Oxidative Stress and Maintaining Cellular Metabolism
Source: Cells. 2022 Feb 9;11(4):599. doi: 10.3390/cells11040599 (PMC8870193; doi:10.3390/cells11040599)
Supplement: Supplementary file 1 [file cells-11-00599-s001.zip › cells-1567584-supplementary.pdf]

Article

Flavonoids from Sacred Lotus Stamen Extract Slows Chronological Aging in Yeast Model by Reducing Oxidative Stress and Maintaining Cellular Metabolism

Duangjai Tungmunnithum [1,2,3,*], Samantha Drouet [2] and Christophe Hano [2,3,*]

[1] Department of Pharmaceutical Botany, Faculty of Pharmacy, Mahidol University, Bangkok 10400, Thailand
[2] Laboratoire de Biologie des Ligneux et des Grandes Cultures, INRAE USC1328, Campus Eure et Loir, Orleans University, 28000 Chartres, France; samantha.drouet@univ-orleans.fr
[3] Le Studium Institue for Advanced Studies, 1 Rue Dupanloup, 45000 Orléans, France
* Correspondence: duangjai.tun@mahidol.ac.th (D.T.); hano@univ-orleans.fr (C.H.)

Supplementary Materials

Table S1. Quantification of the major flavonoids of the *N. nucifera* stamen extract.

	Concentration (mg/100g DW)
Myr-3-Glc	5.94 ± 0.23
Rut	4.68 ± 0.14
Que-3-Glu	3.21 ± 0.09
Kae-3-Rob	8.99 ± 0.21
Kae-3-Glc	10.81 ± 0.36
Kae-3-Glu	16.05 ± 0.34
Iso-3-Glc	4.09 ± 0.17

Mean ± standard deviations (n = 3). Myr: myricetin; Myr-3-O-Glc: Myr-3-O-glucoside; Que: quercetin; rut : rutinoside; rutin: Quer-3-O-Rut; Quer-3-O-Glu: Quer-3-O-glucuronic acid; Kae: kaempferol; Kae-3-O-Glc: Kae-3-O-glucoside; Kae-3-O-Rob: Kae-3-O-robinobioside; Kae-3-O-Glu: Kae-3-O-glucuronic acid; Iso: isorhamnetin; Iso-3-O-Glc: Iso-3-O-glucoside.

Table S2. *In vitro* antioxidant capacity of the *N. nucifera* stamen.

Activity	Stamen extract
DPPH [1]	183.69 ± 4.84
ABTS [1]	60.45 ± 2.80
FRAP [1]	319.60 ± 13.14

[1] antioxidant capacity expressed in µmol TEAC (trolox equivalent antioxidant activity); DPPH: 2,2-diphenyl-1-picrylhydrazyl; ABTS: 2,2-azinobis (3-ethylbenzthiazoline-6-sulphonic acid; FRAP: ferric reducing antioxidant power.

Table S3. Viability of yeast cells under the different treatment conditions determined 48 h after treatment.

	viability
CTL	95.37 ± 2.61 [a]
RES	94.24 ± 2.01 [a]
LES 0.25	96.59 ± 1.91 [a]
LES 0.5	95.83 ± 1.45 [a]
LES 1.0	95.80 ± 3.67 [a]

LSE: sacred lotus stamen extract (at 0.25, 0.5 and 1.0 mg/mL, respectively). RES: E-Resveratrol (10 μM) used as positive antiaging control. Values are means ± standard deviations (SD) of 4 independent experiments. Different letters represent significant differences between the different conditions ($p < 0.05$).

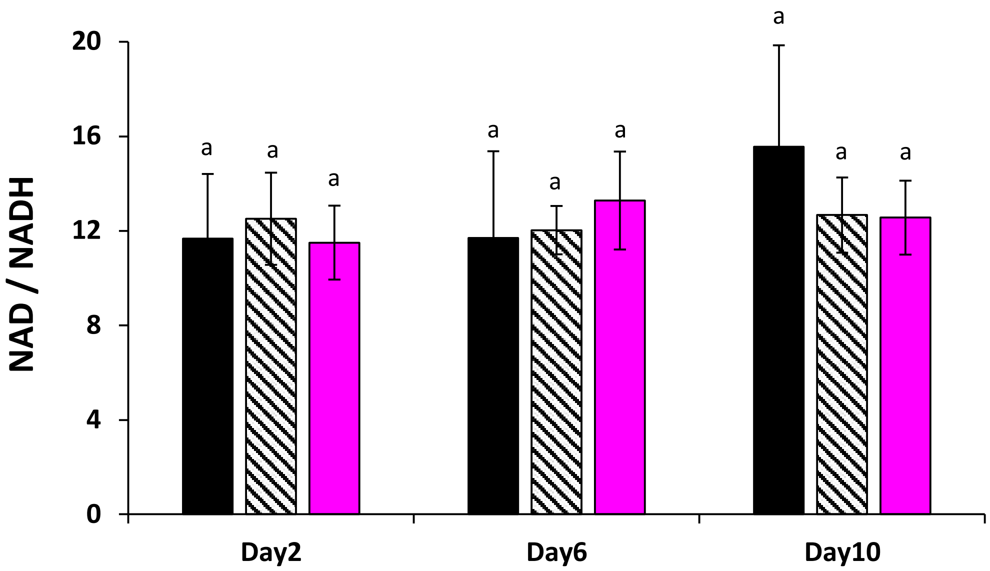

Figure S1. Impact of LSE on NAD/NADH ratio. LSE: sacred lotus stamen extract (0.5 mg/mL). RES: *E*-Resveratrol (10 μM) used as positive antiaging control. Significant differences ($p < 0.05$) are shown by different letters.

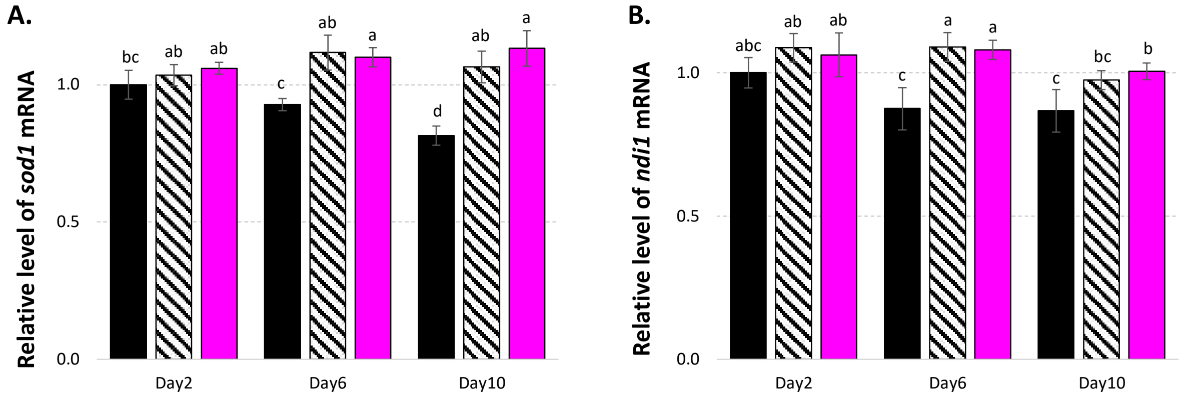

Figure S2. Effects of LSE on A. *SOD1* and B. *NDI-1* gene expression. LSE: sacred lotus stamen extract (0.5 mg/mL). RES: E-Resveratrol (10 μM) used as positive antiaging control. Significant differences ($p < 0.05$) are shown by different letters.
